# The Impact of Moderate Chronic Hypoxia and Hyperoxia on the Level of Apoptotic and Autophagic Proteins in Myocardial Tissue

**DOI:** 10.1155/2018/5786742

**Published:** 2018-08-16

**Authors:** Alexandra Gyongyosi, Laura Terraneo, Paola Bianciardi, Arpad Tosaki, Istvan Lekli, Michele Samaja

**Affiliations:** ^1^Department of Pharmacology, Faculty of Pharmacy, University of Debrecen, Debrecen, Hungary; ^2^Department of Health Science, University of Milan, Milan, Italy

## Abstract

The redox imbalance and the consequent oxidative stress have been implicated in many pathological conditions, including cardiovascular diseases. The lack or the excess of O_2_ supply can alter the redox balance. The aim of the present study was to understand the heart responses to prolonged hypoxia or hyperoxia and how such situations may activate survival mechanisms or trigger cell death. Seven-week-old Foxn1 mice were exposed to hypoxia (10% O_2_), normoxia (21% O_2_), or hyperoxia (30% O_2_) for 28 days, then the heart tissue was excised and analyzed. The alterations in redox balance, housekeeping protein levels, and autophagic and apoptotic process regulation were studied. The D-ROM test demonstrated an increased oxidative stress in the hypoxic group compared to the hyperoxic group. The level of hypoxia inducible factor-1 (HIF-1*α*) was increased by hypoxia while HIF-2*α* was not affected by treatments. Chronic hypoxia activated the biochemical markers of autophagy, and we observed elevated levels of Beclin-1 while LC3B-II and p62 were constant. Nevertheless, we measured significantly enhanced number of TUNEL-positive cells and higher Bax/Bcl2 ratio in hyperoxia with respect to hypoxia. Surprisingly, our results revealed alterations in the level of housekeeping proteins. The expression of *α*-tubulin, total-actin, and GAPDH was increased in the hypoxic group while decreased in the hyperoxic group. These findings suggest that autophagy is induced in the heart under hypoxia, which may serve as a protective mechanism in response to enhanced oxidative stress. While prolonged hypoxia-induced autophagy leads to reduced heart apoptosis, low autophagic level in hyperoxia failed to prevent the excessive DNA fragmentation.

## 1. Introduction

The mammal heart is an organ with relatively high demand for O_2_ and high O_2_ consumption; the O_2_ supply may often be insufficient with respect to needs due to limitations in O_2_ delivery, thereby establishing a condition of hypoxia that may frequently accompany cardiovascular disorders such as thrombosis, atherosclerosis, and pulmonary disorders [1]. Being strictly associated with ischemia, hypoxia represents one of the leading causes of death worldwide [2]. Reactive oxygen species (ROS) are known to play crucial roles in the functional response of the myocardium to altered O_2_ levels [3], and their role has been extensively investigated [4]. One of the most intuitive therapies against hypoxia, that is, O_2_ therapy or hyperoxia, may however be associated with O_2_ toxicity and oxidative stress, and breathing mixtures with >40% O_2_ markedly elevates ROS release [5]. Excess O_2_ is also expected to alter the expression of those genes that regulate the recruitment of cell mechanisms in response to varied O_2_ levels. Intuitively, the direction of the alteration is opposite to that elicited by the reverse condition, hypoxia. Such responses might be considered to have a relevant impact on the body's adaptation to altered O_2_ consumption, which includes the regulation of gene transcription by the hypoxia-inducible factors (HIFs). HIFs act as transcriptional factors for an array of genes that participate in various cell processes such as angiogenesis, metabolism, cell proliferation, and control of ROS-induced damage [6], including apoptosis and autophagy [7, 8]. Traditionally, HIFs are believed to help in the adaptation to low O_2_ tension, but recent studies have demonstrated that HIF-1*α* and HIF-2*α* are upregulated in response to hyperoxia as well, at least in the brain [9], some types of cancer [10], and hepatocytes [11].

The present study was designed to compare the responses to chronic moderate hypoxia and hyperoxia in the heart. We avoided extreme hypoxia/hyperoxia conditions for three reasons: (1) to prevent the involvement of nonspecific, potentially confounding signals; (2) to simulate as closely as possible the clinical conditions of hypoxic patients and patients in O_2_ therapy; and (3) to employ evenly spaced O_2_ levels (10%–21%–30% O_2_) to help in the quantitative comparison in the responses to the two stresses. In addition, we selected a chronic (4 weeks) condition to assess the involvement of gene-based mechanisms that are recruited to establish, or at least try to establish, myocardial adaptation to altered O_2_ levels. One difficulty encountered in this study was the effect of hypoxia/hyperoxia on the level of the housekeeping proteins normally employed as loading controls in Western blot analyses, thereby introducing a considerable bias in the quantitative assessment of the target proteins. Therefore, we dedicated effort to get alternative reliable loading controls. The aim of the present work is to establish whether altered O_2_ levels in the inspired atmosphere have divergent effects on a few key myocardial responses to stress, with particular concern to apoptosis, autophagy, and some other factors responsible for cardioprotection.

## 2. Materials and Methods

### 2.1. Animals and Treatments

The animals used in the present study (*n* = 18) were male seven-week-old Foxn1 mice (Harlan Laboratory) with an average weight of 27–30 g. They were acclimatized to a 12 h light/12 h dark cycle and housed at an ambient temperature of 25 ± 2°C. Food and water were freely accessible ad libitum. All animals were cared in accordance to the Guide for the Care and Use of Laboratory Animals [12], and the use of the animals was approved by the University of Milan Committee for the Use of Laboratory Animals (OBPA).

The mice were randomly transferred into a gas chamber flushed as described earlier [9]. The animals were randomly segregated into three different groups as follows: 10% O_2_ (hypoxia, *n* = 6), 21% O_2_ (normoxia, *n* = 6), and 30% O_2_ (hyperoxia, *n* = 6). The animals were sacrificed and the hearts were harvested at the end of 28 days of exposure. Briefly, the mice were transferred into the compensation chamber, anesthetized by i.p. Na-thiopental (10 mg/100 g body weight) and heparin (500 unites) by subcutaneous injection. Next, following induction of deep anesthesia, chest cavities were opened and the hearts were excised, frozen in liquid nitrogen, and stored at −80°C for later analyses.

### 2.2. Hemoglobin Level and D-ROM Test

Blood hemoglobin concentration was measured by the Drabkin method, assuming *ε* = 11.05 cm^−1^ mM^−1^ [10]. To evaluate the oxidative stress, we determined the overall level of oxidant chemical species produced, including ROS, H_2_O_2_, and hypochlorous acid. By attacking organic molecules, these species generate stable reactive O_2_ metabolites (ROMs), primarily composed of hydroperoxides (ROOH). To determine oxidative stress in plasma, we used the photometric D-ROM test (Diacron International srl, Grosseto, Italy) that evaluates the capacity of in vivo-formed ROOH to generate alkoxyl (•R-O) and peroxyl (•R-OO) radicals in the presence of iron released from plasma by an acidic buffer. Data are expressed as mg H_2_O_2_/dL.

### 2.3. Immunofluorescent Cell Death Detection

Frozen samples were embedded in optimum cutting temperature (OCT-Compound, Leica Instruments, Nussloch, Germany), and cut into 5 *μ*m-thick sections by cryomicrotome (Leica CM1510) at −22°C. All tissue sections were placed on Superfrost Plus glass slides (Thermo Scientific, Rockford, IL) and dried at room temperature for 2 min. To detect apoptosis, we used the terminal deoxynucleotidyl transferase (Tdt) nick end labelling test by the In Situ Cell Death Detection Kit, TMR (fluorescein-labeled cell markers) red (Roche, Mannheim, Germany). Apoptosis can be detected by labeling the free 3′-OH termini with modified nucleotides in an enzymatic reaction. The enzyme Tdt catalyzes the template-independent polymerization of deoxyribonucleotides to the 3′-end of single- and double-stranded DNA.

The sections were fixed in 4% cold-buffered formalin, at 4°C for 45 min, then rinsed two times for 5 min in PBS (phosphate-buffered saline) pH 7.4, at 4°C. Next, they were postfixed in ethanol-acetic acid (2 : 1, *v*/*v*) at −20°C for 5 min and washed twice for 5 min in PBS. The sections were boiled in citrate buffer pH 6.0 for 12 min, then cooled at room temperature for 20 min, thereafter washed two times for 5 min in PBS. Finally, the sections were incubated with Tdt in a humidified box, at 37°C for 1 hour. After washing, to identify nuclei, we used the blue karyophilic dye Hoechst 32258 (Sigma Aldrich, Schnelldorf, Germany). Glycerol + PBS pH 8.0 was used as mounting medium. Confocal microscopic images were obtained by confocal microscope (Leica SP2 confocal microscope with He/Kr and Ar lasers; Heidelberg, Germany). After merging the blue and red channels, purple spots were associated with apoptotic nucleus, while blue spots were identified as nonapoptotic nucleus (Adobe Photoshop CC 2017, San Jose, CA, USA). Apoptosis was quantified by ratio of Tdt-positive nuclei/total nuclei in each section.

### 2.4. Western Blot Analysis

Frozen heart tissue (50–80 mg) was lysed in a glass potter in a 1 : 3 ratio (*w*/*v*) buffer A (10 mM HEPES, 1.5 mM MgCl_2_, 0.5 mM DTT, 0.2 mM PMSF, 10 mM KCl, and 10% protease inhibitor cocktail (Roche, Mannheim, Germany), pH 7.9). Homogenates were kept on ice for 20 min and centrifuged at 14000 rpm at 4°C for 20 min. The pellet was resuspended and centrifuged again for 10 min at 14000 rpm. Supernatants resulting from the two centrifugations were merged, transferred to a new tube, and used as cytosolic extract. The resultant pellet was resuspended in isolating buffer B (20 mM HEPES, 1.5 mM MgCl_2_, 0.5 mM DTT, 0.2 mM PMSF, 420 mM NaCl, 0.2 mM EDTA, 25% glycerol, and 10% protease inhibitor cocktail (Roche, Mannheim, Germany), pH 7.9), kept on ice for 20 min, and centrifuged at 14000 rpm at 4°C for 20 min. The protein concentration was determined by a Coomassie Protein Assay Kit (Thermo Scientific, Rockford, IL) using bovine serum albumin as the standard. Samples were mixed with loading buffer and boiled for 5 min.

A total of 70 *μ*g of protein in each sample was loaded and separated on 8–15% SDS-PAGE gels (Sigma Aldrich, Schnelldorf, Germany) and then blotted onto a nitrocellulose membrane (PerkinElmer Life and Analytical Sciences, Boston, MA). After blocking the membranes with 5% nonfat dry milk in Tris-buffered saline with 0.1% Tween 20 (TBST), the membranes were incubated overnight with primary antibody solution. Subsequently, the membranes were washed with TBST 3 times and incubated with the respective horseradish peroxidase- (HRP-) conjugated secondary antibody solution for 1 h at room temperature. The following primary antibodies and dilutions were used: anti-*α*-tubulin (Santa Cruz Biotechnology, 1 : 1000), anti-actin (Sigma Aldrich, St Louis, 1 : 2000), anti-GAPDH (Sigma Aldrich, St Louis, 1 : 15000), anti-Beclin-1 (Epitomics, Abcam Company, 1 : 3000), anti-LC3B (Cell Signaling Technology, 1 : 1000), anti-p62 (Abcam, 1 : 1000), anti-HIF-1*α* (Santa Cruz Biotechnology, 1 : 300), anti-HIF-2*α* (Abcam, 1 : 300), anti-Akt (Cell Signaling Technology, 1 : 1000), anti-phospho-Akt-Ser^473^ (Cell Signaling Technology, 1 : 1000), anti-Bcl_2_ (Santa Cruz Biotechnology, 1 : 1000), anti-Bax (Santa Cruz Biotechnology, 1 : 500), anti-AMPK (Santa Cruz Biotechnology, 1 : 1000), anti-phospho-AMPK-Thr^172^ (Santa Cruz Biotechnology, 1 : 1000), and anti-NOX4 (Abcam, 1 : 5000). The secondary antibodies were used: peroxidase-conjugated AffiniPure goat anti-rabbit IgG (H + L) (Jackson ImmunoResearch Laboratories Inc., 1 : 10000) and peroxidase-conjugated AffiniPure goat anti-mouse IgG (H + L) (Jackson ImmunoResearch Laboratories Inc., 1 : 10000). After washing, the membranes were incubated with Lite Ablot chemilumisencence substrate (Lite Ablot, Euro Clone, EMPO10004) to visualize by enhanced chemiluminescence bands according to the recommended procedure (UVITEC Ltd., Cambridge, UK). The band intensities were measured by UVI-1D software.

### 2.5. Western Blot Analysis with Stain-Free Gels

A total of 70 *μ*g of protein in each sample was loaded and separated on TGX Stain-Free™ 7.5% acrylamide gels (Bio-Rad Laboratories, USA). Then, gels were exposed to UV light, thereby trihalo compounds of the stain-free gels covalently bind to tryptophan residues of the proteins. Subsequently, the proteins were blotted onto a nitrocellulose membrane (PerkinElmer Life and Analytical Sciences, Boston, MA, USA). After transfer, the proteins of the membrane were exposed by another brief irradiation, and the resulting tryptophan adducts emitted fluorescence signal. This picture was evaluated and used as total protein volume. The further steps were performed in the same way described under Section 2.4. The chemiluminescent bands and each total protein lane intensities were measured by UVI-1D software. In this method, protein density and quantification are measured directly on the Western blot membranes with reference to total loaded proteins. This type of normalization totally eliminates the need to select an adequate panel of housekeeping proteins [13]. The protein expression was quantified by the ratio of (band volume)/(total protein volume).

### 2.6. Statistical Analyses

All data passed the Kolmogorov-Smirnov normality test (*α* = 0.05). Two types of analysis were performed to compare the groups. In the first, we considered three independent groups (hypoxia, normoxia, and hyperoxia) and performed the one-way analysis of variance (ANOVA), followed by the Tukey multiple comparison test if ANOVA *P* < 0.05. In the second, data were considered as a continuous function of % O_2_ (10, 21, and 30), and we performed linear regression analysis, followed by the generation of the best-fit equations. To assess the goodness of the fit, we calculated the squared correlation coefficient (*r*2) to generate the *P* value of the regression (http://vassarstats.net/rsig.html). If *P* < 0.05, we assessed whether the slope of the best-fit line was significantly different from zero, that is, there is a statistical effect of the O_2_ level in the inspired atmosphere on the variable under study. Statistical analyses were performed using Prism (GraphPad Software Inc.).

## 3. Results

### 3.1. Body Weight and Blood Hemoglobin

**Figure 1:**
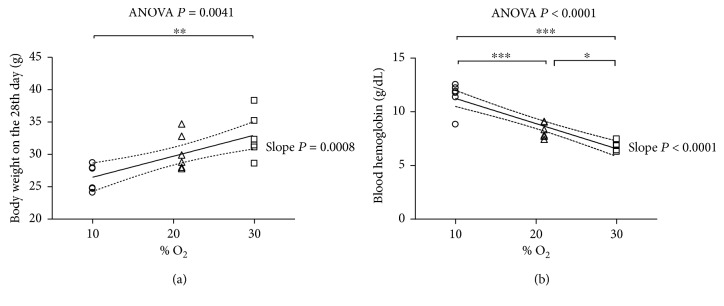
Changes in (a) body weight and (b) blood hemoglobin after exposure to 10% O_2_, 21% O_2_, and 30% O_2_ for 28 days (*n* = 6, 6, and 6, resp.). Blood hemoglobin concentration was measured by Drabkin's method. The linear regression yielded *r* ^2^ = 0.8259 (*P* < 0.0001) and when significant *P* < 0.05 is displayed with the 95% CI (dotted curves) and the slope *P*. The ANOVA test *P* value is reported in the figure. When significant *P* < 0.05, ^∗^, ^∗∗^, and ^∗∗∗^ represent *P* < 0.05, *P* < 0.01, and *P* < 0.001 at the Tukey posttest, respectively.

All mice survived the various treatments. The initial body weight (BW) of animals at time zero was 27.8 ± 0.6 g (mean ± SEM) with no statistical differences among the groups.  Figure 1 and Supplementary Figure 1 show the BW at the 28th day of exposure to 10%, 21%, or 30% O_2_. Although the Tukey test did not show statistical differences between 21% O_2_ and 10% O_2_, as well as between 21% O_2_ and 30% O_2_, the linear regression analysis shows that the slope was significantly greater than zero, indicating that the BW values were positively related to % O_2_.

Treatments with different % O_2_ in breathed air differentially altered the blood hemoglobin level. Whereas breathing low % O_2_ elevated the blood hemoglobin level, high % O_2_ lowered it. This parameter was strictly dependent on % O_2_, as shown by the inverse linear regression relationship between blood hemoglobin and % O_2_ (*P* < 0.0001, Figure 1(b) and Supplementary Figure 1(b)).

### 3.2. Oxidative Stress

**Figure 2:**
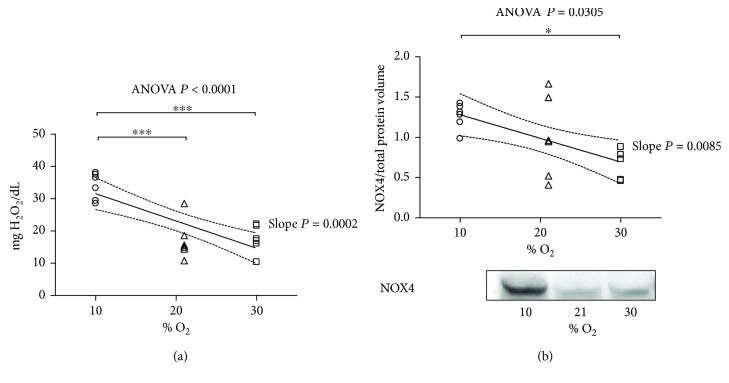
Oxidative stress at the end of the exposure to 10% O_2_, 21% O_2_, and 30% O_2_ for 28 days. (a) D-ROM (reactive O_2_ metabolite) test to estimate the level of oxidant species in plasma, expressed in mgH_2_O_2_/dL (*n* = 6, 6, and 6, resp.). (b) The expression level of NADPH oxidase subunit 4 (NOX4) (*n* = 6, 6, and 5, resp.) and representative picture. The inset reports strong, inverse linear correlation; (a) the linear regression yielded *r* ^2^ = 0.5900, correlation coefficient (*r*) = 0.768, *P* value = 0.0002; (b) the linear regression yielded *r* ^2^ = 0.2817, correlation coefficient (*r*) = 0.53, *P* value = 0.023 and when significant *P* < 0.05 is displayed with the 95% CI (dotted curves) and the slope *P*. The ANOVA test *P* value is reported in the figure. When significant *P* < 0.05, ^∗^ and ^∗∗∗^ represent *P* < 0.05 and *P* < 0.001 at the Tukey posttest, respectively.

The D-ROM test enables the determination of the concentration of reactive O_2_ metabolites in biological samples.  Figure 2(a) and Supplementary Figure 2(a) show the level of reactive O_2_ metabolites in the plasma. The value found in the 21% O_2_ group was considered as a normal level [14]. A significant increment was observed in samples obtained from animals kept in 10% O_2_, indicating that the systemic redox balance under hypoxic condition is markedly impaired. However, the hyperoxic environment did not alter the systemic prooxidant pool compared to 21% O_2_. Nevertheless, we observed strong and statistically significant inverse association between the level of oxidant species and the O_2_ concentration (*P* = 0.0002).

Nicotinamide adenine dinucleotide phosphate (NADPH) oxidase subunit 4 (NOX4) acts as an O_2_ sensor, catalyzes the reduction of molecular O_2_ to various reactive O_2_ species (ROS), and plays an important role in cardiovascular pathophysiology. We found an elevated expression of NOX4 in 10% O_2_ in comparison with 30% O_2_, indicating increased oxidative stress (Figure 2(b) and Supplementary Figure 2(b)). Moreover, we observed inverse linear relationship between the level of NOX4 and % O_2_ (*P* = 0.03).

### 3.3. Hypoxia Signaling

**Figure 3:**
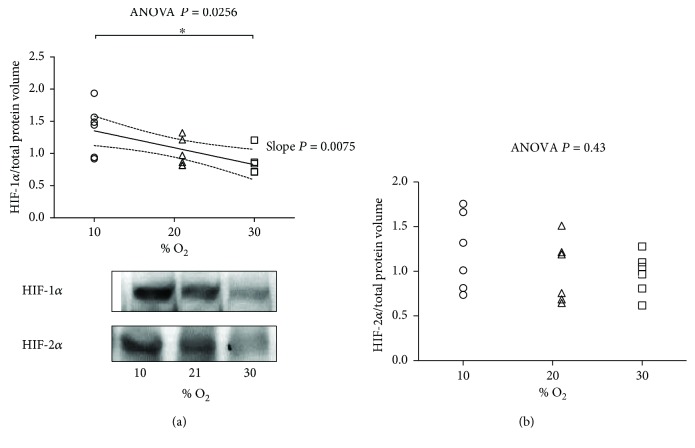
Hypoxia signaling: (a) expression level of hypoxia-inducible factor- (HIF-)1*α* after exposure to 10% O_2_, 21% O_2_, and 30% O_2_ for 28 days (*n* = 6, 6, and 5, resp.) and representative picture; (b) expression level of hypoxia-inducible factor- (HIF-) 2*α* (*n* = 6, 6, and 6, resp.). The inset reports high, inverse linear correlation; (a) the linear regression yielded *r* ^2^ = 0.3886, correlation coefficient (*r*) = 0.623, *P* value = 0.0075 and when significant *P* < 0.05 is displayed with the 95% CI (dotted curves) and the slope *P*. (b) no linear relationship. The ANOVA test *P* value is reported in the figure. When significant *P* < 0.05, ^∗^ represent *P* < 0.05 at the Tukey posttest.

We measured the expression of HIF-1*α* and HIF-2*α* in cardiac cytosolic extracts by Western blot, with the loading control represented by the total protein volume as explained in Section 2.5 (Figure 3 and Supplementary Figure 3). The HIF-1*α* protein level was upregulated in 10% O_2_ compared to 30% O_2_ as for the significant difference between 10% O_2_ and 31% O_2_ at the ANOVA and Tukey tests. Furthermore, a linear correlation is observed (*P* = 0.0075) for HIF-1*α*. By contrast, there is no significant effect of neither 10% O_2_ nor 30% O_2_ on the expression patterns of HIF-2*α*.

### 3.4. DNA Fragmentation and Apoptosis

**Figure 4:**
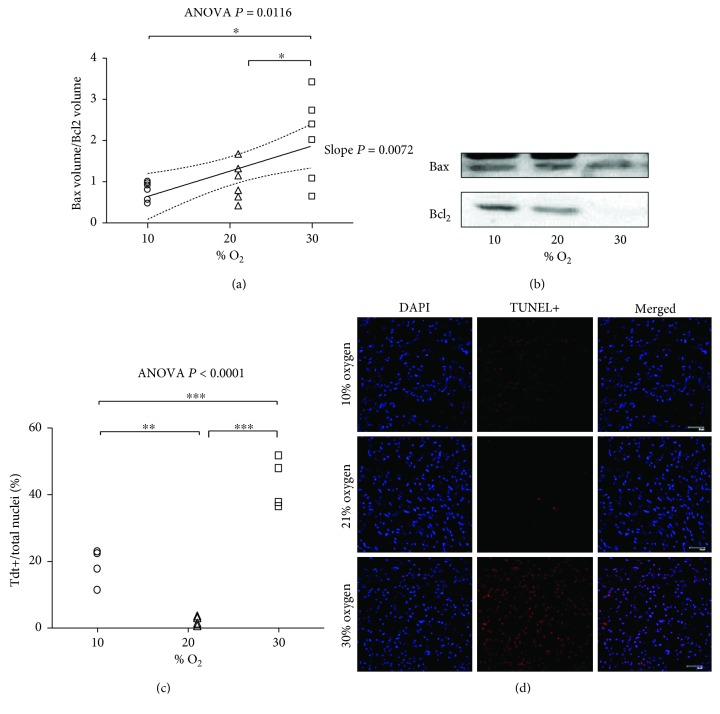
DNA fragmentation and apoptosis: (a) expression level of the ratio of Bax to Bcl_2_ after exposure to 10% O_2_, 21% O, and 30% O_2_ for 28 days (*n* = 6, 6, and 6, resp.); (b) representative picture of Bax and Bcl_2_; (c) results of TUNEL assay, percent of the ratio of Tdt+ to total nuclei (*n* = 4, 4, and 4, resp.). The inset reports high, positive linear correlation; (a) the linear regression yielded *r* ^2^ = 0.3723, correlation coefficient (*r*) = 0.61, *P* value = 0.0072 and when significant *P* < 0.05 is displayed with the 95% CI (dotted curves) and the slope *P*. (c) The inset report the one-way ANOVA and Tukey multiple comparison post hoc test. ^∗^ *P* < 0.05; ^∗∗^ *P* < 0.01; ^∗∗∗^ *P* < 0.001. (d) Representative immunofluorescence pictures obtained in heart tissue samples from hypoxia (10% O_2_), normoxia (21% O_2_), and hyperoxia (30% O_2_) for 28 days.

To evaluate the effect of % O_2_ in breathed air on cardiomyocyte apoptosis, we first analyzed the Bax/Bcl_2_ ratio, which determines the cell susceptibility to apoptosis. As this representation uses the ratio of two spots in the same gel, the bias introduced by the instability of the housekeeping proteins is avoided. The results show upregulated Bax/Bcl_2_ ratio in 30% O_2_ compared to both 10% O_2_ (*P* = 0.05) and 21% O_2_ (*P* = 0.05, Figure 4(a) and Supplementary Figure 4(a)). Direct detection of apoptotic nuclei by the immunofluorescent staining TUNEL technique, however, gave a different result. The percentage of apoptotic nuclei in 10% O_2_, 21% O_2_, and 30% O_2_ was 18.6 ± 2.7, 2.3 ± 0.7, and 43.4 ± 3.8 (mean ± SEM), respectively, indicating that, whereas very low in normoxia, moderate hypoxia markedly increases the degree of Tdt-positive nuclei, but moderate hyperoxia induces a still higher degree of fragmented DNA (Figures 4(c), 4(d), and Supplementary Figure 4(b)). Therefore, the percentage of Tdt-positive nuclei was not linearly related with % O_2_, but rather a U-shaped relationship was observed.

### 3.5. Effects of Different O_2_ Concentrations on Activation of Survival Pathways

**Figure 5:**
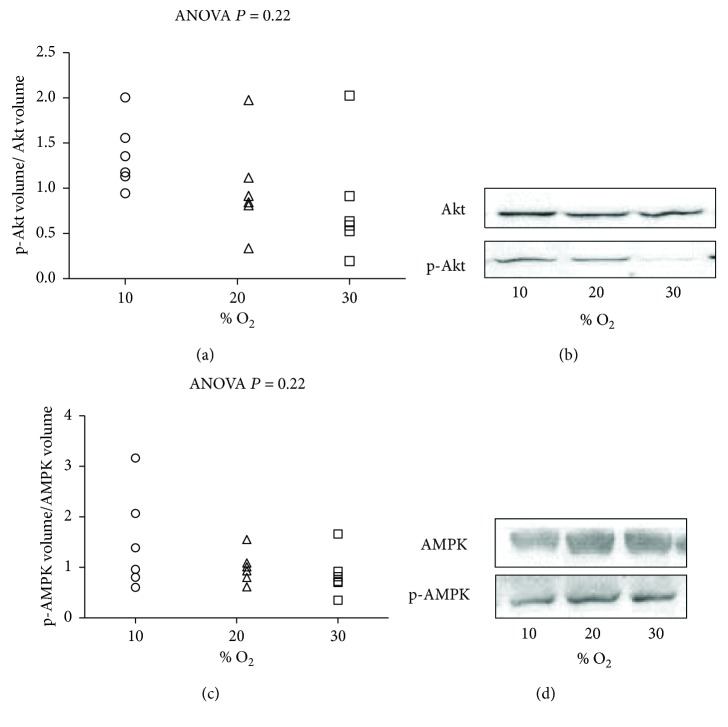
Survival pathways after exposure to 10% O_2_, 21% O_2_, and 30% O_2_ for 28 days (*n* = 6, 6, and 6, resp.). (a) The expression level of the ratio of p-Akt to Akt; (b) representative picture of Akt and p-Akt. (c) The expression level of the ratio of p-AMPK to AMPK in heart tissue samples from hypoxia (10% O_2_), normoxia (21% O_2_), and hyperoxia (30% O_2_) for 28 days. (d) Representative picture of AMPK and p-AMPK. The inset reports linear correlation, (a, c) no linear relationship. The ANOVA test *P* value is reported in the figure.

Akt is a serine/threonine kinase that has been shown to play a central role in promoting cell survival and opposing apoptosis.  Figure 5(a) and Supplementary Figure 5(a) report that % O_2_ was not related to this pathway. The ratio of total Akt expression and the phosphorylated form at Ser^473^ (p-Akt) remained unaltered in the groups.

We investigated another relevant defense mechanism which is often activated by stress. AMP-activated protein kinase (AMPK) is an energy sensor activated by increases in (AMP) or by oxidant stress (reactive O_2_ species (ROS)). Similarly, no alteration was observed in the ratio of p-AMPK/AMPK between the groups. (Figure 5(c) and Supplementary Figure 5(b)). There was no statistically significant correlation between the activation of investigated survival pathways and alteration of O_2_ concentration.

### 3.6. Consequence of Modified O_2_ Tension on Autophagy

**Figure 6:**
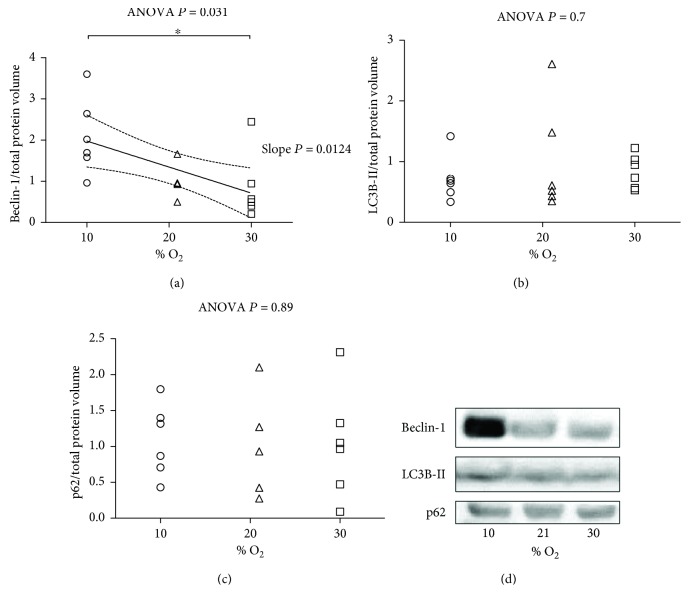
Autophagy markers after exposure to 10% O_2_, 21% O_2_, and 30% O_2_ for 28 days (*n* = 6, 6, and 6, resp.). (a) The expression level of Beclin-1. The inset reports strong, inverse linear correlation; (a) the linear regression yielded *r* ^2^ = 0.3501, correlation coefficient (*r*) = 0.591, *P* value = 0.0124 and when significant *P* < 0.05 is displayed with the 95% CI (dotted curves) and the slope *P*. (b) The expression level of LC3B-II, no linear relationship. (c) The expression level of p62, no linear relationship. The ANOVA test *P* value is reported in the figure. When significant *P* < 0.05, ^∗^ represent *P* < 0.05 at the Tukey posttest. (d) Representative picture of Beclin-1, LC3B-II, and p62.

We assessed autophagy by examining the expression of several autophagy proteins. As part of a type III phosphoinositide-3 kinase complex, the autophagy gene Beclin-1 is required for the formation of the autophagic vesicles. As shown in Figure 6(a) and Supplementary Figure 6(a), the protein expression level significantly increased in 10% O_2_ compared to 30% O_2_. Besides this, the strong and inverse linear relationship between the level of Beclin-1 and % O_2_ was detected (*P* = 0.012). Interestingly, the average level of Beclin-1 autophagic protein was similar in 30% O_2_ and 21% O_2_, indicating that the autophagy pathway was not induced by O_2_.

The level of LC3B-II and p62 proteins was not altered after the treatment of different % O_2_ in breathed air (Figures 6(b) and 6(c) and Supplementary Figures 6(b) and 6(c)). These proteins were not statistically associated with the modified O_2_ tension.

### 3.7. Housekeeping Proteins

**Figure 7:**
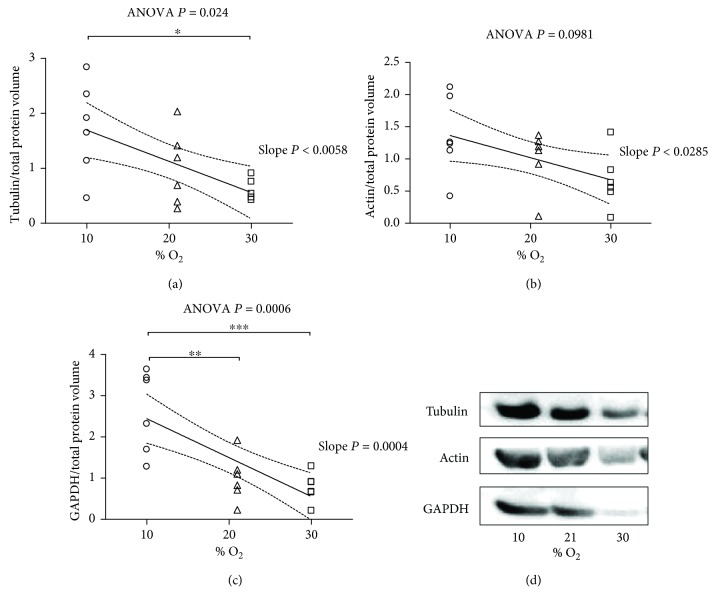
Housekeeping proteins after exposure to 10% O_2_, 21% O_2_, and 30% O_2_ for 28 days (*n* = 6, 6, and 6, resp.). (a) The expression level of *α*-tubulin. (b) The expression level of actin. (c) The expression level of glyceraldehyde 3-phosphate dehydrogenase (GAPDH). The inset reports strong, inverse linear correlation; (a) the linear regression yielded *r* ^2^ = 0.3874, correlation coefficient (*r*) = 0.622, *P* value = 0.0058; (b) the linear regression yielded *r* ^2^ = 0.2658, correlation coefficient (*r*) = 0.515, *P* value = 0.0285; (c) the linear regression yielded *r* ^2^ = 0.5518, correlation coefficient (*r*) = 0.742, *P* value = 0.0004 and when significant (*P* < 0.05) is displayed with the 95% CI (dotted curves) and the slope *P*. The ANOVA test *P* value is reported in the figure. When significant *P* < 0.05, ^∗^, ^∗∗^, and ^∗∗∗^ represent *P* < 0.05, *P* < 0.01, and *P* < 0.001 at the Tukey posttest, respectively. (d) Representative picture of *α*-tubulin, actin, and GAPDH.

Figure 7 and Supplementary Figure 7 show the effect of varying % O_2_ in inspired air on the tissue density of three housekeeping proteins (*α*-tubulin, actin, and GAPDH) as determined by Western blot analyses. In these experiments, the level of the target proteins is expressed with reference to the total protein volume. Surprisingly, the Tukey test shows that 30% O_2_ induced higher expression of *α*-tubulin and GAPDH, but not actin, than 10% O_2_. However, the slope of the best-fit line was significantly less than zero for all the three housekeeping proteins, showing that higher % O_2_ in inspired air corresponds to decreased expression of these proteins (*P* = 0.006, 0.03, and 0.0004, resp.).

All of representative pictures can be find in Supplementary Materials.

## 4. Discussion

In this study, we examined the myocardial response to 28-day normobaric hypoxia, normoxia, or hyperoxia. In the described experimental model, confounding phenomena related to reoxygenation or deoxygenation events are prevented [15]. The choice of the % O_2_ in the three groups was dictated by two orders of considerations. First, we wanted to use clinically relevant O_2_ levels avoiding poorly significant extremely hypoxic or hyperoxic situations. The % O_2_ selected for hypoxia is known to induce sublethal metabolic and signaling changes in the myocardial tissue [16–18]. The % O_2_ selected for hyperoxia mimics a situation common in pulmonary patients who breathe with the aid of portable O_2_ concentrators [19] and has been shown to induce important signaling in the brain tissue [9]. Second, we wanted to use evenly spaced % O_2_ in a linear progression (10–21–30) to enable the detection of linear correlations with % O_2_. Of interest, the redox imbalance did not appear pronounced in hyperoxia but was greater in hypoxia. In a previous study, the D-ROM levels in rats exposed to 14 to 82% O_2_ for 24 h indicated that the redox imbalance occurs only for >40% O_2_ [5]. Here, we found that no alteration was evident in rats exposed to 30% O_2_ for one month.

### 4.1. Hypoxia Signaling

HIFs are heterodimeric transcription factors, consisting of an *α* and a *β* subunit [20]. The expression of the *α*-subunit is directly regulated by the O_2_ level. Three different HIF-*α* subunits are known with variable tissue expressions and abundances but similar O_2_-dependent mechanisms of regulation [21, 22]: HIF-1*α*, which occurs in all nucleated cells and is responsible for acute responses; HIF-2*α*, which is expressed in the brain tissue, vascular endothelium, and type II pneumocytes, responsible for chronic responses; and HIF-3*α*, which is found in parts of the brain, lung, heart, liver, and kidney [22, 23], but the function is not yet clear. Similarly, our results show that chronic hypoxia (10% O_2_) induces the expression of HIF-1*α* in the heart tissue. Furthermore, in accordance with the literature and our HIF-1*α* results, as an underlying mechanism, an elevated oxidative stress was found in the hypoxic group.

In rats exposed to 50% O_2_ for 3 weeks, both HIF-1*α* and HIF-2*α* accumulate in the brain during the first week of exposure, followed by a progressive decline [24]. In mice exposed to 30% O_2_ for 28 days, neurons display marked increase of nuclear HIF-2*α*, unrelated to oxidative stress [9]. These findings in the brain tissue are indirectly confirmed in other organs and tissues as growing prostate cancer [25], as well as newborn rat hepatocytes and liver hematopoietic cells [26]. Newborn rat hearts were exposed for 2 weeks to 60% O_2_ and 95% O_2_ hyperoxia; the HIF-1*α* nuclear translocation increased significantly upon 95% O_2_ exposure and remained unaltered at lower O_2_ tension [27]. Additionally, Zara et al. reported an increased percentage of apoptotic cells in the same group, which can be due to the trigger ROS-mediated membrane injury [27]. In contrast to these studies, after considering the experimental conditions used in this study, where we did not find any relevant hyperoxia-induced increase in neither HIF-1*α* nor HIF-2*α*, it is plausible to assume that 30% O_2_ is not a sufficient trigger for HIFs in the heart.

### 4.2. DNA Fragmentation and Apoptosis

Apoptosis, as programmed cell death, is characterized by distinct morphological characteristics and energy-dependent biochemical mechanisms. Membrane blebbing, phosphatidylserine flip-flop, chromatin condensation, protein and DNA fragmentation, and cell shrinkage are hallmarks of cells under apoptosis [28]. It was reported that HIF-1 seems to be involved in initiating apoptosis. When cells are exposed to chronic hypoxia, the overexpressed HIF-1 in alveolar epithelial cells resulted in increased apoptosis [29].

A direct link was demonstrated between HIF-1*α* and proapoptotic members of the Bcl-2 subfamily such as Nip3, which is likely a direct target gene for HIF-1*α* [30]. On the other hand, the p53 tumor suppressor protein can activate target genes which initiates cell death via Bax or causes growth arrest in response to stress or DNA damage. Various physiological processes lead to HIF-1*α*-dependent p53-mediated induction of apoptosis in hypoxia [31].

The TUNEL assay is a method commonly used to assess the cell entry into the apoptotic pathway, although it fails in discriminating apoptosis from necrosis and autolytic cell death [32]. DNA fragmentation is common to different kinds of cell death, and in the liver, for example, apoptosis and necrosis in vivo occur asynchronous [33], although DNA fragmentation in apoptosis is more pronounced [32, 34]. Our TUNEL assay indicated elevated DNA fragmentation under hypoxic and hyperoxic conditions, although Tdt-positive nuclei were more enhanced under hyperoxia. It was correlated with HIF-1*α* under hypoxia. On the other hand, Bax/Bcl_2_ ratio was significantly increased after hyperoxia. This may reflect the fact that DNA fragmentation and apoptosis were more pronounced under hyperoxia.

### 4.3. Autophagy

As an evolutionary highly conserved [35] catabolic self-degradative process that is important for balancing energy sources [36], autophagy may enhance cell survival [37] or be a way for cell death. Autophagy is triggered by different stimuli such as hypoxia [38], hyperoxia [39], and oxidative stress [40] or during energy imbalance [41]. Autophagy and apoptosis often occur in the same cell, mostly in a sequence where autophagy precedes apoptosis. While autophagy allows cell adaptation to stress, massive autophagy may instead favor death [42]. The induction of autophagy requires the autophagy-related genes (Atgs), which are involved in maturation and recycling of autophagosomes [43]. Acute (48 h) mimic hypoxia in H9c2 cardiomyoblast cells increases apoptosis, accumulates autophagosome, and increases the expression of autophagy-related protein such as LC3-II, ATG5, and Beclin-1, likely through BNIP3 (BH3-only protein) [44]. BNIP3 protein can disrupt the inhibitory interaction between Beclin-1 and Bcl_2_ [45]. ROS is a powerful activator of autophagy in the myocardial tissue during reperfusion [46, 47], and ROS scavengers may protect the heart from ischemia/reperfusion injury through autophagy regulation [48]. Prolonged hyperoxia (95% O_2_, 72 hours) increases autophagosome formation and expression level of LC3B in lung epithelial cells [49]. However, the data reported here show only significant increase in the expression of Beclin-1 under hypoxia. Furthermore, Beclin-1, which initiates the autophagy process, was not altered even in 30% O_2_, and we failed to observe any significant alteration in the levels of LC3B and p62. However, we did not measure the autophagic flux, and therefore we cannot draw any definitive conclusion on the relation between autophagy and high % O_2_ in the heart, mainly in hyperoxia. The question whether a possible crosstalk between apoptosis and autophagy exists may be raised. Based on previous findings [50], it is attractive to speculate that Beclin-1 may be induced by the oxidative stress upon prolonged moderate hypoxia and might alleviate the elevation of Bax/Blc_2_ ratio, which could give a room for adaptation. Nevertheless, under prolonged hyperoxia, relative lack of oxidative stress might fail to increase Beclin-1 leading to enhanced DNA fragmentation.

### 4.4. Housekeeping Proteins

At the beginning of the present investigation, traditional Western blot was employed. Unexpectedly, the level of the reference proteins that are generally used to normalize densitometry values were subjected to considerable fluctuations. Employing total protein normalization methods confirmed the suspicion that the level of these commonly used housekeeping proteins fluctuated as a function of inspired % O_2_. Our results suggested that the expression of housekeeping proteins was remarkably increased by hypoxia (10% O_2_) treatment. In line with these surprising observations, studies performed in human failing myocardium and under other circumstances highlighted significant alterations in GAPDH [51, 52], actin, and tubulin [53, 54]. Likewise, investigations in housekeeping gene expression changes in diffuse alveolar damage induced by hyperoxia (90–95% O_2_ for 1–3 days) in a mouse lung revealed increased GAPDH protein at maintained GAPDH mRNA levels [55]. Taken together, our unexpected finding reported here suggests that data normalized against housekeeping proteins should be handled with caution when the inhaled O_2_ tension is altered.

## 5. Conclusion

The present study suggests that at varying inhaled O_2_ tensions, the level of the commonly used housekeeping proteins such as *α*-tubulin, actin, and GAPDH may be altered. Thus, these proteins should be used with caution as loading controls, and the alternative method to normalize densitometry values against total protein content is to be taken into serious consideration. Furthermore, 10% O_2_ appears to cause a considerable level of oxidative stress in the heart tissue, which increases the expression of HIF-1*α*, while 30% O_2_ does not cause significant oxidative stress as evidenced by unaltered level of derivatives of ROMs. However, both hypoxia and hyperoxia elevate TUNEL positivity (Table 1). Alterations in the Beclin-1 protein level suggest that altered O_2_ tensions may have an impact on autophagy. Although a definitive conclusion cannot be drawn because the autophagy flux was not measured, a crosstalk between apoptosis and autophagy may have been established under these circumstances.

### 5.1. Limitations of the Study

Although the hypoxia/hyperoxia model was described elsewhere [9], here we used the D-ROM test, hemoglobin levels, and NOX-4 expression to examine the body's response to O_2_ level alterations, neglecting other markers such as plasma lactate, other redox-related enzymes, and especially heart size, which could help to draw a more complex picture on the body's response to altered O_2_ tension. Despite the markers used here that are commonly used to monitor macroautophagy, we did not measure the autophagic flux [56] which may be useful to further assess the effects of hypoxia/hyperoxia on autophagy.

## Figures and Tables

**Table 1:** Effects of moderate, chronic hypoxia, and hyperoxia on different variables.

	Hypoxia	Hyperoxia
Body weight	↓	↑
Blood hemoglobin	↑	↓
D-ROMs	↑	↔
NOX4	↑	↔
HIF-1*α*	↑	↔
HIF-2*α*	↔	↔
Bax/Bcl_2_	↔	↑
DNA fragmentation	↑	↑
p-Akt	↔	↔
p-AMPK	↔	↔
Beclin-1	↑	↔
LC3B-II	↔	↔
p62	↔	↔

## Data Availability

All data used to support the findings of this study are available from the first author upon request.

## Supplementary Materials

Supplementary Materials Results are presented as bar graphs in Supplementary Figures 1–7. Furthermore, full representative blots are presented in Supplementary Figure 8.
