# Supplementary material for: The Impact of Moderate Chronic Hypoxia and Hyperoxia on the Level of Apoptotic and Autophagic Proteins in Myocardial Tissue
Source: Oxid Med Cell Longev. 2018 Aug 16;2018:5786742. doi: 10.1155/2018/5786742 (PMC6116398; doi:10.1155/2018/5786742)

# The impact of moderate chronic hypoxia and hyperoxia on the level of apoptotic and autophagic proteins in myocardial tissue

Alexandra Gyongyosi^a^, Laura Terraneo^b^, Paola Bianciardi^b^, Arpad Tosaki^a^, Istvan Lekli^a^, Michele Samaja^b^

^a^ Department of Pharmacology, Faculty of Pharmacy, University of Debrecen, Hungary
^b^ Department of Health Science, University of Milan, Italy

Running title: Altered oxygen tension and cell death

Keywords
heart; hypoxia; hyperoxia; apoptosis; autophagy

**Corresponding Author:**

Istvan Lekli, Ph.D.

Dept. Pharmacology, Faculty of Pharmacy

University of Debrecen

Nagyerdei krt. 98

4032 – Debrecen, Hungary

Phone/Fax: 36-52-255586

email: [lekli.istvan@pharm.unideb.hu](mailto:lekli.istvan@pharm.unideb.hu)

# Supplementary Figure legends

**Supplementary Figure 1:** Changes in (A) body weight and (B) blood hemoglobin after exposure to 10 % O_2_, 21 % O_2_ and 30 % O_2_ for 28 days (n=6, 6, and 6, respectively). Bar graph shows Mean and Standard deviation. Blood hemoglobin concentration was measured by the Drabkin's method. The ANOVA test P value is reported in the figure. When significant (P<0.05), *, **, and *** represent P<0.05, P<0.01, and P<0.001 at the Tukey’s post-test, respectively.

**Supplementary Figure 2:** Oxidative stress at the end of the exposure to 10 % O_2_, 21 % O_2_ and 30 % O_2_ for 28 days. Bar graph shows Mean and Standard deviation. (A) D-ROMs (Reactive O_2_ Metabolites) test to estimate the level of oxidant species in plasma, expressed in mgH_2_O_2_/dL (n=6, 6, and 6, respectively). (B) Expression level of NADPH oxidase subunit 4 (NOX4) (n=6, 6, and 5, respectively) and representative picture. The ANOVA test P value is reported in the figure. When significant (P<0.05), *, **, and *** represent P<0.05, P<0.01, and P<0.001 at the Tukey’s post-test, respectively.

**Supplementary Figure 3:** Hypoxia signaling. Bar graph shows Mean and Standard deviation. (A) Expression level of hypoxia-inducible factor (HIF)-1α after exposure to 10 % O_2_, 21 % O_2_ and 30 % O_2_ for 28 days (n=6, 6, and 5, respectively) and representative picture. (B) Expression level of hypoxia-inducible factor (HIF)-2α (n=6, 6, and 6, respectively). The ANOVA test P value is reported in the figure. When significant (P<0.05), *, **, and *** represent P<0.05, P<0.01, and P<0.001 at the Tukey’s post-test, respectively.

**Supplementary Figure 4:** DNA fragmentation and apoptosis. Bar graph shows Mean and Standard deviation. (A) Expression level of the ratio of Bax to Bcl_2_  after exposure to 10 % O_2_, 21 % O_2_ and 30 % O_2_ for 28 days (n=6, 6, and 6, respectively), (B) Results of TUNEL assay, percent of the ratio of Tdt+ to total nuclei (n=4, 4, and 4, respectively). The inset report the One-way ANOVA and Tuckey multiple comparison post hoc test. *, P<0.05; **, P<0.01; ***, P<0.001

**Supplementary Figure 5**: Survival pathways after exposure to 10 % O_2_, 21 % O_2_ and 30 % O_2_ for 28 days (n=6, 6, and 6, respectively). Bar graph shows Mean and Standard deviation. (A) Expression level of the ratio of p-Akt to Akt, (B) Expression level of the ratio of p-AMPK to AMPK in heart tissue samples from hypoxia (10 % O_2_), normoxia (21 % O_2_) and hyperoxia (30 % O_2_) for 28 days. The ANOVA test P value is reported in the figure. When significant (P<0.05), *, **, and *** represent P<0.05, P<0.01, and P<0.001 at the Tukey’s post-test, respectively.

**Supplementary Figure 6:** Autophagy markers after exposure to 10 % O_2_, 21 % O_2_ and 30 % O_2_ for 28 days (n=6, 6, and 6, respectively). Bar graph shows Mean and Standard deviation. (A) Expression level of Beclin-1. (B) Expression level of LC3B-II. (C) Expression level of the ratio of LC3B-II and LC3B-I. (D) Expression level of p62, no linear relationship. The ANOVA test P value is reported in the figure. When significant (P<0.05), *, **, and *** represent P<0.05, P<0.01, and P<0.001 at the Tukey’s post-test, respectively

**Supplementary Figure 7:** Housekeeping proteins after exposure to 10 % O_2_, 21 % O_2_ and 30 % O_2_ for 28 days (n=6, 6, and 6, respectively). Bar graph shows Mean and Standard deviation. (A) Expression level of α-tubulin. (B) Expression level of actin. (C) Expression level of glyceraldehyde 3-phosphate dehydrogenase (GAPDH). The ANOVA test P value is reported in the figure. When significant (P<0.05), *, **, and *** represent P<0.05, P<0.01, and P<0.001 at the Tukey’s post-test, respectively. (D) Representative picture of α-tubulin, actin, and GAPDH.

**Supplementary Figure 8:** Representative pictures for each Western blot results: NOX4, HIF-1α, HIF-2α, Bax, Bcl_2_, p-AMPK, AMPK, p-Akt, Akt, Beclin-1, LC3B-II, p62, Tubulin, Actin, GAPDH respectively.

**Supplementary figures**

**Supp. Figure 1.**

**a**

**b**

**Supp. Figure 2.**

**a**

**b**

**Supp. Figure 3.**

**a**

**b**

**Supp. Figure 4.**

**a**

**b**

**Supp. Figure 5.**

**a**

**b**

**Supp. Figure 6.**

**a**

**b**

**c**

**d**

**Supp. Figure 7.**

**a**

**b**

**c**

**Supp. Figure 8.**

HIF-2

216
CH

369
N

381
HO

222
CH

370
N

374
HO

219
CH

214
N

380
HO

286
CH

273
N

377
HO

279
CH

212
N

384
HO

220
CH

371
N

375
HO

PK
Brain


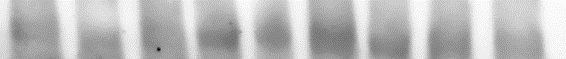

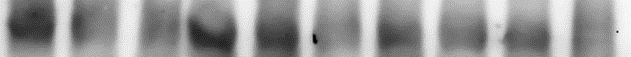

Supplement: Supplementary Materials — Results are presented as bar graphs in Supplementary Figures 1–7. Furthermore, full representative blots are presented in Supplementary Figure 8. [file 5786742.f1.docx]
